# Biochar and Seed Priming Technique with Gallic Acid: An Approach toward Improving Morpho-Anatomical and Physiological Features of *Solanum melongena* L. under Induced NaCl and Boron Stresses

**DOI:** 10.1021/acsomega.3c01720

**Published:** 2023-07-26

**Authors:** Shumaila, Sami Ullah, Wadood Shah, Aqsa Hafeez, Baber Ali, Shahid Khan, Sezai Ercisli, Abdullah Ahmed Al-Ghamdi, Mohamed S. Elshikh

**Affiliations:** † Department of Botany, University of Peshawar, Peshawar 25120, Pakistan; ‡ Biological Sciences Research Division, Pakistan Forest Institute, Peshawar 25120, Pakistan; § Department of Plant Sciences, Quaid-i-Azam University, Islamabad 45320, Pakistan; ∥ Crops, Environment and Land Use Programme, Crop Science Department, Teagasc, Carlow R93 XE12, Ireland; ⊥ Department of Horticulture, Agricultural Faculty, Ataturk Universitesi, 25240 Erzurum, Turkey; # HGF Agro, Ata Teknokent, 25240 Erzurum, Turkey; ∇ Department of Botany and Microbiology, College of Science, King Saud University, Riyadh 11451, Saudi Arabia

## Abstract

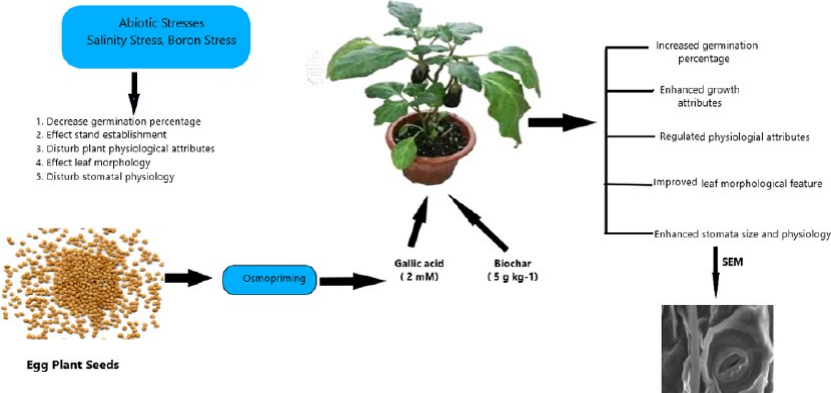

Dynamic shifts in climatic patterns increase soil salinity and boron levels, which are the major abiotic factors that affect plant growth and secondary metabolism. The present study assessed the role of growth regulators, including biochar (5 g kg^–1^) and gallic acid (GA, 2 mM), in altering leaf morpho-anatomical and physiological responses of *Solanum melongena* L. exposed to boron (25 mg kg^–1^) and salinity stresses (150 mM NaCl). These growth regulators enhanced leaf fresh weight (LFW) (70%), leaf dry weight (LDW) (20%), leaf area (LA), leaf area index (LAI) (85%), leaf moisture content (LMC) (98%), and relative water content (RWC) (115%) under salinity and boron stresses. Physiological attributes were analyzed to determine the stress levels and antioxidant protection. Photosynthetic pigments were negatively affected by salinity and boron stresses along with a nonsignificant reduction in trehalose, GA, osmoprotectant, and catalase (CAT) and ascorbate peroxidase (APX) activity. These parameters were improved by biochar application to soil and presoaking seeds in GA (*p* < 0.05) in both varieties of *S. melongena* L. Scanning electron microscopy (SEM) and light microscopy revealed that application of biochar and GA improved the stomatal regulation, trichome density, epidermal vigor, stomata size (SS) (13 381 μm), stomata index (SI) (354 mm^2^), upper epidermis thickness (UET) (123 μm), lower epidermis thickness (LET) (153 μm), cuticle thickness (CT) (11.4 μm), trichome density (TD) (23 per mm^2^), vein islet number (VIN) (14 per mm^2^), vein termination number (VTN) (19 per mm^2^), midrib thickness (MT) (5546 μm), and TD (27.4 mm^2^) under salinity and boron stresses. These results indicate that the use of inexpensive and easily available biochar and seed priming with GA can improve morpho-anatomical and physiological responses of *S. melongena* L. under oxidative stress conditions.

## 1 Introduction

Climate change is a current global issue and is estimated to increase the mean global temperature by 1.0–5.7 °C at the end of the 21st century.^1−3^ Changing climate patterns negatively affect crop growth and yield,^4,5^ with adverse effects on global food production.^6−11^ A variety of biotic^12−14^ and abiotic stresses^15−17^ created by environmental conditions include salinity,^18,19^ drought,^20−22^ temperature,^23^ floods, and heavy-metal stress.^24−28^ Salinity stress affects approximately 20% of irrigated land globally.^29,30^ It negatively affects the photosynthesis rate by inhibiting photochemical routes via reduction in stomata size (SS), stomatal closing, inhibiting nutrient uptake,^31−33^ and disrupting water balance.^34,35^ High levels of salts in the soil decrease stomatal conductivity, which further restricts the inward movement of CO_2_ and thus disrupts gaseous exchange.^36^ This subsequently interrupts electron transport chain reactions and decreases the photosynthesis rate.^37−40^ Salinity stress increases the trichome density (TD) and leaf size and adversely affects the plant height, total leaf area, and stomatal density.^41^ Salinity disrupts different physiological and biochemical functions of plants.^29,42,43^ For example, NaCl toxicity stimulates oxidative stress via formation of reactive oxygen species (ROS) that result in lipid peroxidation and damage to biomolecules.^44−46^

In addition to salinity stress, arid and semi-arid areas are subject to boron stress.^47,48^ Ferguson et al.^49^ characterized the toxic effect of boron stress that causes leaf injuries in pistachio plants. Boron stress decreases total dry mass and flower bud formation,^49^ reduces shoot growth, and damages the cell wall.^50^ Sang et al.^51^ reported that the toxic effect of high boron levels is due to alterations of protein biosynthesis and carbohydrate metabolism. Boron reacts with some basic metabolites and creates stable complexes that cause physiological impairment inside cells and disrupts plant growth and development.^52^ Boron also damages cell membranes via formation of ROS, which cause cell and tissue damage.^53^ In response to osmotic stress, plants synthesize compatible solutes such as soluble protein, soluble sugars, trehalose sugar (TS), carotenoids, proline, and antioxidant enzymes, such as superoxide dismutase (SOD), peroxidase (POD), ascorbate peroxidase (APX), and catalase (CAT), that detoxify ROS.^54−56^ Boron stress potentiated salt toxicity in tomato and cucumber plants. Excess of boron in the soil imposed extracellular pressure on photosynthetic activity and limits the growth and yield of tomato and spinach under salt stress.^57^

Biochar is a biomass of carbon, a low-cost porous pyrogenous matter formed by heating organic waste^58,59^ (such as crop deposits or animal manure) under zero or inadequate oxygen environments in a closed furnace at elevated temperatures ≤700 °C through pyrolysis.^60^ Biochar is extensively used due to its appropriate carbon content in nutrient-poor and degraded soils. Biochar provides minerals and nutrients (such as Mg, S, Ca, K, and P) and improves soil physical status (such as bulk density, aggregate stability, high cation exchange capacity [CEC], porosity, and saturated hydraulic conductivity).^61^ In detail, it increased microbial biomass carbon in the saline soil and the activities of urease, invertase, and phosphatase in bulk soils and rhizosphere soils under maize cultivation. When applied in saline soils, composted biochar increased the soil organic matter content and CEC and decreased the exchangeable Na and soil pH.^62^ Thus, enhancing soil chemical and biological characteristics subsequently increases crop antioxidant defense mechanisms, yield, and microbial activity and reduces leaching of nutrients.^63,64^ Similarly, among different allelochemicals, phenolic compounds not only act as efficient free-radical scavengers but also inhibit the lipid peroxidation process, stabilize cell membranes, and act as cell defense system against ROS.^19,65,66^ Gallic acid (GA; 3,4,5-trihydroxybenzoic acid) is extracted from many plants and has antioxidant potential.^67^ GA was used in maintaining crop capacity to improve the growth rate and photosynthetic ability under induced abiotic stress in *Glycine max* L.^68^ GA induced tolerance in rice seedlings against NaCl stress by enhancing the activities of H_2_O_2_-scavenging enzymes, such as POD, SOD, CAT, and APX, and thus protected cell membranes from oxidative damage caused by ROS and lipo-peroxide accumulation.^69^

Eggplant (*Solanum melongena* L.) is the second most valuable crop of the family Solanaceae. *S. melongena* L. is widely grown and consumed in Southeast Asia and in the southern parts of Pakistan.^70^ Globally, the cultivation area of *S. melongena* L. is approximately 1.86 million hectares.^71^ In Pakistan, eggplant occupies an area of 9044 ha with an average yield of 88 148 tons ha^–1^.^72^ In Pakistan, most agricultural practices take place in arid and semi-arid areas in the warm season, with low precipitation and in soils with high salt content. Scanning electron microscopy (SEM) and UV–vis spectrophotometry are extensively applied tools for investigating the surface features of plant leaves, including the morphology of stomata, trichomes, stomatal density, epidermal characteristics, cuticular layer, quantification of nanoparticles, infection, and physicochemical components.^73^

The objectives of this study were to utilize UV–vis spectrophotometry, SEM, and light microscopy tools and energy-dispersive X-ray (EDX) to characterize leaf physicochemical components, leaf surface, cross-sectional anatomy, and elemental composition and to examine the responses of growth stimulators by activating natural defense systems both enzymatically and nonenzymatically under stress conditions. Currently, there is no published scientific report on the possible preventive roles of gallic acid and biochar on individual and combined effect of salinity and boron stresses in eggplant. In addition, there is no scientific report present on anatomical features and agronomic and physio-biochemical attributes of eggplant under such induced abiotic stressors and its amelioration through biochar and gallic acid treatment.

## 2 Materials and Methods

### 2.1 Biochar Preparation and Physicochemical Analysis by SEM and EDX

**Figure 1:**
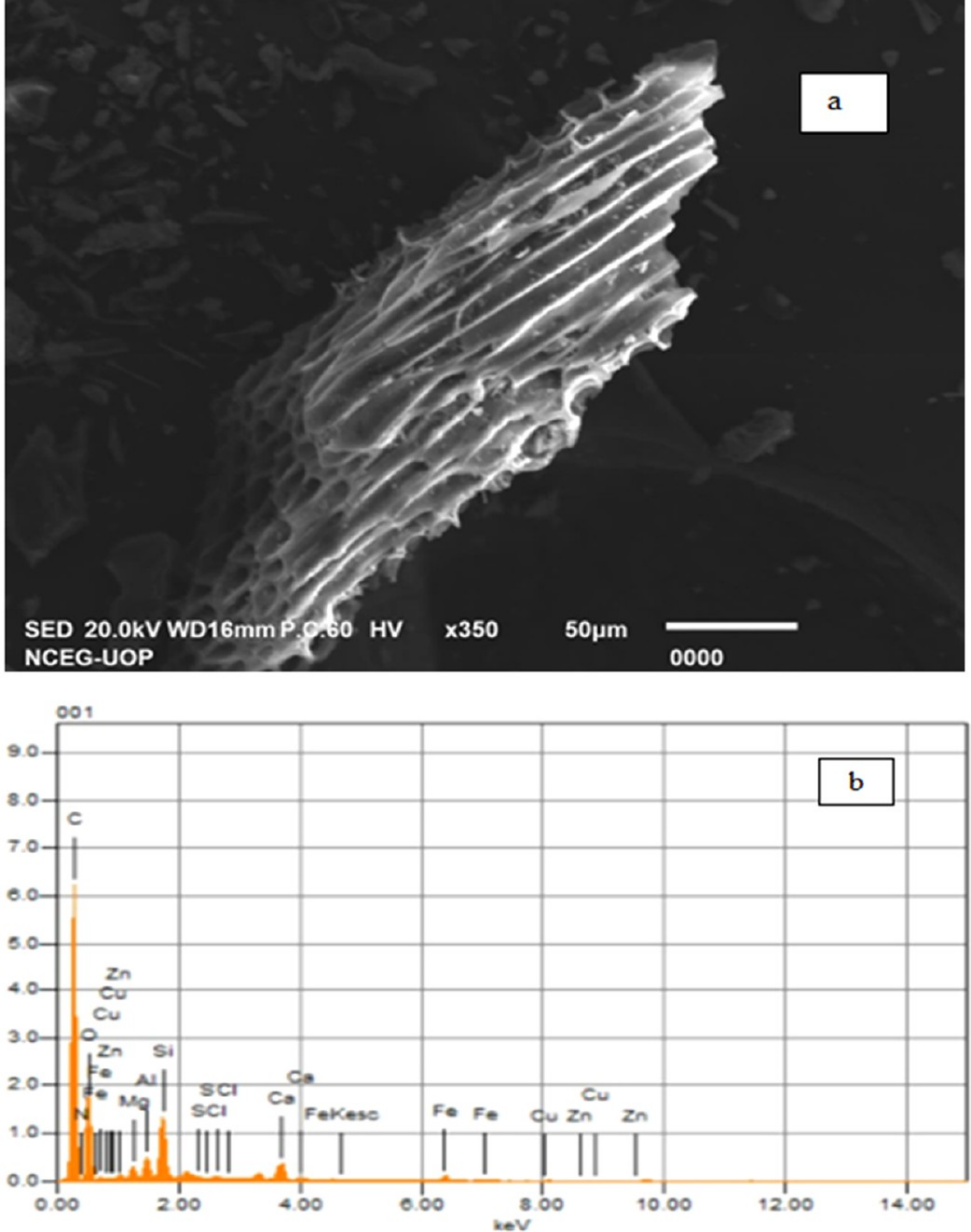
Morphological structure and elemental analysis of biochar obtained through (a) scanning electron microscopy and (b) energy-dispersive X-ray analysis.

Biochar used in the experiment was produced from hardwood of *Vachellia nilotica* L. in traditional kilns with domestic charcoal. The kiln internal temperature was maintained at 500–550 °C observed with a thermocouple for 24–48 h of pyrolyzing. Prior to application, the produced biochar was air-dried, crushed, and sieved through a 2 mm sieve. Well-powdered biochar was analyzed with exposure to gold glaze placed on Spi coating segments for morphological characteristics using a scanning electron microscope (JSMIT100-JEOL-JAPAN) according to the methodology of Lalay et al.^74^ The biochar had large pores and rod-shaped morphology with sharp cracks; this unique structure has the capacity of water holding, araciality of carbon content in soil environments, and invites more microbes, including fungi, ascomycetes, and algae that improve soil fertility. The electrical conductivity (EC) of saturated soils (6.7 dS m^–1^) was measured at 25 °C by a calibrated EC meter (BANTE, DDS-12DW, China). Energy-dispersive X-ray spectroscopy (INCA200/Oxford instruments, U.K.) was used to determine EC (6.7 dS m^–1^), bulk density (0.48 g cm^–3^), cation exchange capacity (5.1 cmol kg^–1^), and perform elemental analysis, including maximum carbon (56.33%), oxygen (35.34%), silicon (3.12%), calcium (1.36%), iron (1.34%), aluminum (1.06%), nitrogen and magnesium (0.63%), chlorine (0.10%), copper (0.06%), zinc (0.04%), and sulfur (0.01%) (Figure 1).

### 2.2 Site Description and Seed Sterilization

Pot experiments were performed at the University of Peshawar (34°1′33.3012″N and 71°33′36.4860″E), KPK Pakistan, during the 2021 growing seasons. The locality of Peshawar lies in the Iranian plateau area with 513 mm of mean annual rainfall. Soil physicochemical studies revealed a silt clay soil texture class as determined by a hydrometer method.^75^ A soil–water suspension (w/v, 1:2.5) was prepared and shaken for 1 h to determine pH with a calibrated pH meter (WTW 7110, Weilheim, Germany). Exchangeable sodium percentage (ESP) of soil (7.5–8.1%) was measured by the method of Page et al.^76^ A soil–water suspension (w/v, 1:2.5) was prepared to determine pH (6.2) with a calibrated pH meter (WTW 7110, Weilheim, Germany). Energy-dispersive X-ray spectroscopy was used to perform soil elemental analysis, including calcium (7.86%), aluminum (7.46%), potassium (2.47%), silicon (20.2%), oxygen (54.86%), iron (6.91%), and zinc (0.20%). The seeds of two varieties (Neelam & BSS 513) of *S. melongena* L. were collected from the National Institute of Food and Agriculture (NIFA), Pakistan. Seeds were surface sterilized with 0.1% mercuric chloride solution, followed by 70% ethanol (5 min) and then washed with deionized water.^77^

### 2.3 Experimental Design and Growth Conditions

Pots were carefully maintained at a nursery in a complete randomized block design (CRBD) at a space of 5 cm away from each other with a net plot size of 3.0 × 2.0 m^2^ for proper air passage. The experiment was performed in a greenhouse in a 2 × 2 × 2 design (two varieties, two levels of abiotic stress-treated and nontreated soil with biochar and seed with GA) between 24/13 °C day/night temperature with an average humidity of 41 and 57% light intensity. Both varieties of eggplant were assessed under 12 treatments in triplicates and were divided into three groups (Table 1). One group served as a control (no stress treatment); in the second group, the seeds were primed with 2 mM GA solution (3,4,5-triphydroxyl-benzoic acid) in 100 mL of water at 4 °C for 1 h^68^ before sowing; in the third group, biochar was mixed at a ratio of 5:1 g kg^–1^ with soil dried for 24 h after sowing. Treatments were designed as the following.

**Table 1:** Experimental Design for Biochar and Seed Priming Technique

treatments	description
T1	control (untreated)
T2	25 mg kg^–1^ boric acid
T3	120 mM NaCl
T4	25 mg kg^–1^ boric acid + 120 mM NaCl
T5	5 g kg^–1^ biochar
T6	5 g kg^–1^ biochar + 25 mg kg^–1^ boric acid
T7	5 g kg^–1^ biochar + 120 mM NaCl
T8	5 g kg^–1^ biochar + 25 mg kg^–1^ boric acid + 120 mM NaCl
T9	2 mM gallic acid
T10	2 mM gallic acid + 25 mg kg^–1^ boric acid
T11	2 mM gallic acid + 120 mM NaCl
T12	2 mM gallic acid + 25 mg kg^–1^ boric acid + 120 mM NaCl

Before seed sowing, 72 pots were well plowed and filled with 2 kg of silt clay (1:2) soil along with farmyard manure. Ten seeds of each variety were sown in earthen pots of 18 cm top and bottom diameter with 20 cm height and placed 10 cm apart in triplicates. Pots were thinned after a week of seed germination, and five healthy seedlings were maintained. Data counted for germination parameters was taken from day 1 to day 10 before applying stress. After 15 days of germination, plants were subjected to salinity stress (120 mM NaCl solution)^69^ or boron stress (boric acid powder of 25 mg kg^–1^ soil).^78^ After induction of stresses on day 25, plants were uprooted and washed with distilled water to remove adhered dust particles for measurements of agronomic properties. After absorbing moisture from the root surface, the fresh weights of root and shoot were measured. The shoot and root lengths and leaf area via dry weights were determined after drying in an oven at 30 °C for 72 h until the weight became constant. Undamaged fresh leaves of plants per treatment in replicates were collected and stored at 4 °C for quantification of photosynthetic pigments, osmoprotectants, and plant antioxidant enzymes via spectroscopy (Spectronic UV-1700, Shimadzu, Japan). Some leaves per treatment were shade-dried for a week and used for morphological studies via SEM. Fresh leaf disks were used for anatomical evaluations by light microscopy.

### 2.4 Determination of Agronomic Characteristics

#### 2.4.1 Relative Water Content (RWC) of Leaves

A fully expanded leaf of three plants per replicate was used to determine the relative water content (RWC) of the leaves by following the standard method of Ogbaga et al.^79^ Three leaf disks of 10 mm were cut by a cork borer through the interveinal area. The fresh weight (*W*_f_) of mean disks was calculated immediately. Preweighed leaf disks were kept in distilled water for 4 h at 20 °C with dim illumination until complete hydration. The fully saturated weight (*W*_s_) of leaf disks was estimated by measuring the dry weight (*W*_d_) for 4 h at 70 °C.
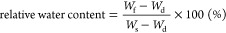
1

#### 2.4.2 Root–Shoot Ratio (RSR)

The dry weight of the shoot and root in all treatments per replicate was measured by the proposed formula of Chuyong and Acidri.^80^
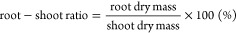
2

#### 2.4.3 Net Assimilation Rate of Leaves

The net assimilation rate (NAR) is the increase in dry mass of a plant per unit time per unit increase in the assimilatory surface. NAR was estimated by the formula of Ghule et al.^81^
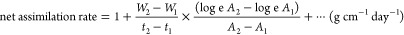
3where *A*_1_ and *A*_2_ are the leaf areas (cm^2^) by the length and width, respectively, while *W*_1_ and *W*_2_ are dry mass in grams at initial time *t*_1_ and final time *t*_2_, respectively.

#### 2.4.4 Leaf Moisture Content (LMC)

Fresh leaves from approximately three per plant in each treatment was taken and weighed. After measuring fresh weight in grams, the leaf was dried in an oven for 72 h at 30 °C and the dry mass was determined. The percent leaf moisture content (LMC) was measured by the proposed formula of Ullah et al.^82^
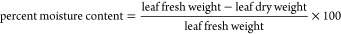
4

#### 2.4.5 Leaf Area Index (LAI)

The leaf width and length were taken randomly from three leaf disks of a single plant per treatment, and the mean leaf area index (LAI) was measured by the formula of Shah et al.^83^
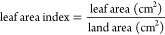
5

#### 2.4.6 Leaf Area Ratio

The leaf area was measured by taking the width and length of three leaf disks per treatment from each plant with a portable leaf area meter (Panomex Inc.) while drying the leaf in an oven for 72 h. The leaf area ratio (LAR) was evaluated following the formula of Shah et al.^83^
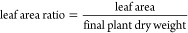
6

#### 2.4.7 Stomata Index (SI)

The stomata index (SI) was calculated by the method of Dubberstein et al.^84^ A cross section of a leaf about 1 mm was cut, its upper epidermis was peeled, and then the section was rinsed with distilled water and 70% ethanol three times. The cell structure of the samples was mounted in Canada balsam, and the number of stomata and epidermal cells was imaged and counted under a Nikon Eclipse E600 microscope (DS-U3, Nikon, Japan). In eq 7, *S* stands for stomata and *E* for epidermal cells.
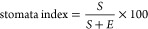
7

### 2.5 Determination of Physiological Components

#### 2.5.1 Quantification of Photosynthetic Pigments

The chlorophyll content in leaves was evaluated by the standard methodology of Zou et al.^85^ Fresh leaves (0.2 g) were ground with a mortar and pestle in 80% acetone and incubated for 24 h in the dark and then centrifuged. The chlorophyll *a* content was determined by measuring absorbance values at 649 nm, chlorophyll *b* content at 663 nm, and carotenoid content at 430 nm. Absorbance was measured with a spectrophotometer against 80% acetone blank.
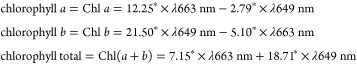


#### 2.5.2 Total Proline Content (TPC)

The total proline content (TPC) of fresh foliar materials was determined following the standard method of Khanam and Mohammad.^86^ A total of 0.5 g of leaves were ground in 10 mL of 3% sulfosalicylic acid with a mortar and pestle. The solution was passed through a Whatman No. 2 filter paper and collected into a test tube. 3% aqueous sulfosalicylic acid was added to make a final volume of 10 mL. 2 mL of glacial acetic acid and acid ninhydrin were mixed in the filtrate. The mixture was boiled for 1 h at 100 °C and then cooled in an ice bath. The reaction was treated with 4 mL of toluene for extraction with vigorous shaking for 15–20 min. The absorbance was measured with a spectrophotometer at 520 nm with red color toluene against a blank toluene. TPC in the sample was calculated using the standard curve for proline in the sample, ranging from 0.1 to 36 μmol on the basis of the fresh mass of the sample. The proline content was expressed in μmol g^–1^ FM.

#### 2.5.3 Determination of Trehalose Sugar

Determination of trehalose sugar (TS) was performed on fresh foliar material according to the standard methodology of Liu et al.^87^ 0.2 g of leaves was crushed with 1 mL of 0.5 M trichloroacetic acid (TCA) in an ice bath, followed by vigorous shaking for 2 h at 0 °C. The mixture was then centrifuged for 10 min at 10 000 rpm, followed by the addition of 0.2 mL of 0.2 N H_2_SO_4_. The solution was then boiled for 10 min at 100 °C and then cooled. Approximately 4 mL of anthrone reagent (0.2 g of anthrone + 100 mL of cold 95% sulfuric acid) was then mixed in the reaction mixture, followed by boiling at 100 °C for 10 min and then cooled. Absorbance was measured with a spectrophotometer at 630 nm.

#### 2.5.4 Determination of Gallic Acid

The GA content was analyzed in fresh leaves by grinding approximately 0.25 g of leaves in 95% methanol. The homogenate was centrifuged at 13 000 rpm for 5 min at 25 °C. The supernatant of 200 mL was then treated with 200 mL of 10% Folin–Ciocalteu (F–C) reagent along with 200 mL of 95% methanol solution. The reaction mixture was treated with 800 mL of 700 mM Na_2_CO_3_ and incubated for 2 h. Optical density (OD) was recorded at 760 nm according to the standard methodology of Yetişsin and Kurt.^67^

#### 2.5.5 Estimation of Catalase Activity (CA)

Catalase activity (CA) was assessed by determining the disappearance of H_2_O_2_ at the initial rate following the method of Khanam and Mohammad.^86^ 0.5 g of fresh leaf was homogenized in 5 mL of buffer solution. 0.1 mL of enzyme extract was added to 3 mL of 30 mM H_2_O_2_ by diluting 0.34 mL of 30% H_2_O_2_ to 100 mL phosphate buffer (pH 7). The decomposition of H_2_O_2_ was followed by decline in optical density (OD) at 240 nm as measured by a spectrophotometer. The interval was 30 s using a blank buffer solution along with the enzyme extract. Enzyme activity was measured in μM H_2_O_2_ kg^–1^ FM s^–1^.

#### 2.5.6 Estimation of Ascorbate Peroxidase Activity

The ascorbate peroxidase assay (APX) was performed according to the method of Salimi et al.^88^ 0.5 g of fresh foliar material was crushed with a mortar and pestle and homogenized in 5 mL of buffer solution. The reaction mixture was then centrifuged at 3000 rpm for 10 min at room temperature. 0.1 mL of enzyme extract was then added to 1.8 mL of 50 mM potassium phosphate (KPO_4_) buffer (pH 7.0), followed by the mixing 0.1 mL of 0.5 mM ascorbic acid solution and 1 mL of 30% H_2_O_2_ in a test tube. Absorbance was measured as the decrease rate of H_2_O_2_ for 3 min at 290 nm optical density against the blank of 30% H_2_O_2_.

### 2.6 Determination of Leaf Anatomy

For leaf analysis, the second oldest fully expanded leaf of a randomly selected plant within each pot was carefully removed using a pair of forceps. One leaf per replicate was harvested and immediately submerged into liquid nitrogen for 1 min, followed by 30 s submersion in a methanol gradient (50, 75, 90, and 100% v/v) and 1 min submersion in hexamethyl disilazane. For SEM analysis, the samples were dried and mounted on SEM cylinder specimen mounts (JSMIT100-JEOL-JAPAN, aluminum, grooved edge, ⌀32 mm). Leaves were oriented such that the adaxial surface could be examined. Mounted leaves were coated with carbon using a JEOL-EC-32010CC coating system for 30 nm. A JEOL scanning electron microscope was used to examine leaf surface anatomy, including epidermis, stomata, and trichomes of each variety at high 20 kV, working distance range 15–16 mm, magnification range 2.20–5.00k×, with a specimen stage *T* = −10 to +90° and *R* = 360°.

The standard methodology of Liu et al.^87^ was used for leaf anatomy studies with light microscopy. On day 15 after salinity and boron stress treatment, mature leaves were randomly obtained from each treatment and cut into small pieces (1 cm × 3 mm) from the region between the main vein to the margin of the leaf and a cross section of midrib with a double-sided blade. Samples were cut carefully to approximately 10 μm thickness and preserved in formalin acetic alcohol solution (90% ethanol, 5% formalin, 5% acetic acid) at 4 °C until dehydration. The cell structure of the samples was mounted in Canada balsam and imaged under a Nikon Eclipse E600 microscope (DS-U3, Nikon, Japan). Leaf pieces from three separate plants per treatment were obtained, and the values were expressed in micrometers. The stomata size, epicuticular thickness, and lamina and midrib thickness (MT) were measured by microscope graticules. Values were the mean of three measurements. The stomata index, vein islet, and vein termination number (VTN) were also recorded in a 1 mm leaf area.

### 2.7 Statistical Analysis

Statistical analysis was a factorial design with induced salinity and boron stress. The analyses were performed in triplicate (*n* = 3) for various parameters, including agronomic, anatomical, physiological, and biochemical attributes and were analyzed by Statistix 10 and IBM SPSS Statistics 22 (SPSS Inc., Chicago IL). Three-way analysis of variance (ANOVA) at significance difference (*p* ≤ 0.05) for all measurements, mean separation, and standard deviations (SD) were compared by Tukey’s multiple range test at *p* ≤ 0.05 for each variety separately. Pearson correlation (*R*) was measured by the same software.

## 3 Results

### 3.1 Determination of Growth Attributes

**Figure 2:**
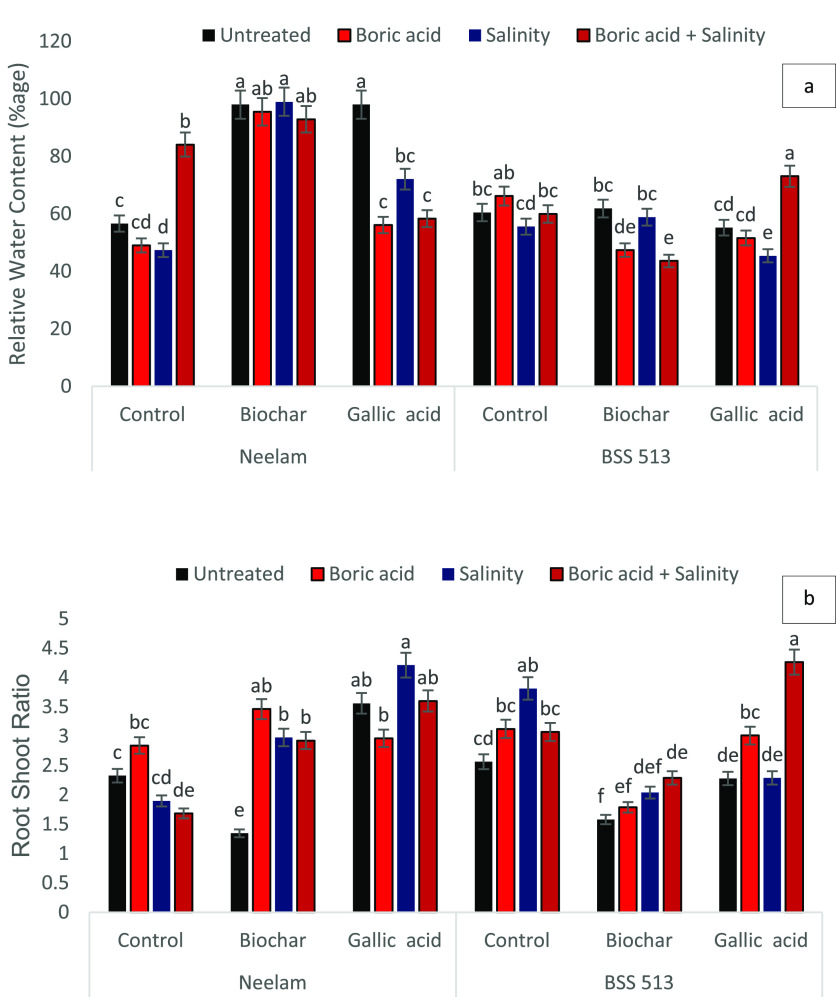
Effects of biochar (5 g kg^–1^) and gallic acid (2 mM) on the (a) relative water content and (b) root–shoot ratio in foliar (mean ± standard) under induced salinity (120 mM NaCl) and boron (25 mg kg^–1^) stress. Vertical bars indicate standard errors with least significance difference among mean values at *p* < 0.05.

Statistical analysis revealed that leaf fresh weight (LFW), leaf dry weight (LDW), and LMC differed with and without the addition of biochar to soil and seed presoaking under abiotic stresses (Table 2). Boron stress reduced LFW (42%), LDW (3.6%), and LMC (18%) nonsignificantly in both varieties. In BSS 513, boron (T10) and salinity stress (T9) reduced LFW and LMC up to 54 and 18%, respectively. A significant increase in these attributes was observed in GA-pre-soaked seeds and after biochar application to soil. With biochar treatment, LFW was increased by 70 and 91%, while LMC was increased by 98 and 86% in both varieties. Likewise, LDW was increased by 12% in Neelam and 31% in BSS 513 with GA-pre-treated seeds under boron stress (T9) and combined abiotic stress (T12). In Neelam, salinity stress with GA-primed seeds (T11) resulted in a significantly reduced leaf area (LA) and LAI by 30 and 16%, respectively, while LAR decreased by 36% under T5. However, a significant (*p* < 0.05) increase in these parameters was observed in GA-pre-soaked seeds under combined abiotic stress. Nonetheless, LA, LAR, and LAI in BSS 513 were decreased by boron stress and combined abiotic stress by 51 and 62%, respectively, without growth regulators and 20% in GA-treated plants. LA and LAI were significantly increased by 85 and 29%, respectively, when soil was treated with biochar (T5) and GA-pre-soaked seeds under salinity stress (T10). LAR increased up to 99%. Furthermore, applied biochar to soil increased leaf RWC significantly (*p* < 0.05) by 115 and 66% in both varieties. GA priming under abiotic stress enhances the ability of biochar to conserve water in soil and thus improve the vigor and viability of plants under stress (Figure 2). Similarly, the root–shoot ratio of plants with respect to abiotic stress was observed after exposure to individual and combined abiotic stress in GA-treated plants in both varieties (Figure 2). However, biochar treatment of soil decreased RSR in Neelam and control untreated (T1) plants of BSS 513. Likewise, no significant difference was measured in leaf NAR of both varieties. ANOVA results through the *F*-ratio (Table 5) represented that all interactions between genotype, treatment, growth regulators, G × T, G × GR, T × GR, and G × T × GR were significant at *p* < 0.05.

**Table 2:** Effect of Biochar (5 g kg^–1^) and Gallic Acid (2 mM) on Growth Indices of *S. melongena* L. under Induced Abiotic Stresses^a^^,^^b^^,^^c^

variety	treatments	LFW	LDW	LMC	LA	LAR	LAI	NAR
Neelam	T1	47 ± 15bcd	12 ± 1.5cde	48.23 ± 9.4bcd	41 ± 5.3de	50 ± 24def	2.32 ± 0.2de	0.0883 ± 0.0a
	T2	42 ± 7.02cd	3.67 ± 3.06f	18.29 ± 20cd	40 ± 2.4de	73 ± 10cde	2.23 ± 0.1de	0.0806 ± 0.0bc
	T3	46 ± 9.29cd	13 ± 6.2cde	67.82 ± 6.7abc	45 ± 2.5de	90 ± 53cde	2.53 ± 0.1de	0.0883 ± 0.0a
	T4	49 ± 10bcd	10 ± 5.1def	32.95 ± 11cd	39 ± 6.0de	43 ± 17efg	2.18 ± 0.3de	0.0827 ± 0.0bc
	T5	70 ± 7.5abc	11 ± 2.0cde	91.84 ± 3.64a	49 ± 3.3cd	36 ± 10.0fg	2.77 ± 0.1cd	0.0839 ± 0.0abc
	T6	44 ± 14.7cd	5.33 ± 5.1ef	65.61 ± 8.7ab	43 ± 4.9de	62 ± 4.2cde	2.40 ± 0.2de	0.0844 ± 0.0ab
	T7	51 ± 25bcd	12 ± 7.9cde	67.72 ± 6.8ab	51 ± 6.0bc	84 ± 52cde	2.84 ± 0.3bc	0.0825 ± 0.0bc
	T8	59 ± 6abcd	10 ± 1.7def	70.09 ± 4.7ab	42 ± 6.8de	53 ± 3.1def	2.34 ± 0.3de	0.085 ± 0.0ab
	T9	52 ± 15bcd	12 ± 6.5cde	55.63 ± 15bc	52 ± 5.9ab	48 ± 11efg	2.92 ± 0.3ab	0.0831 ± 0.0abc
	T10	43.1 ± 20cd	7.6 ± 1.5def	44.54 ± 27bc	42 ± 5.3de	51 ± 13def	2.37 ± 0.2de	0.0812 ± 0.0bc
	T11	38 ± 7.94cd	4.6 ± 1.53ef	74.53 ± 8.4ab	30 ± 26.0e	63 ± 58cde	1.66 ± 1.40e	0.0832 ± 0.0abc
	T12	54 ± 17bcd	10 ± 3.6def	75.07 ± 5.7ab	54 ± 5.2ab	92 ± 72abc	3.02 ± 0.2ab	0.0831 ± 0.0abc
BSS 513	T1	93 ± 10.9ab	36 ± 12.86a	60.24 ± 20bc	81 ± 11.8a	53 ± 14def	4.53 ± 0.60a	0.0835 ± 0.0abc
	T2	80 ± 48abc	11 ± 5.5cde	54.07 ± 22bc	51 ± 7.9bc	96.0 ± 89a	2.88 ± 0.4bc	0.0846 ± 0.0abc
	T3	78±34abc	20 ± 1.5abc	60.56 ± 27bc	81 ± 7.74a	96 ± 22bcd	4.54 ± 0.40a	0.0835 ± 0.0abc
	T4	80 ± 29abc	17 ± 3.6bcd	71.40 ± 1.4ab	61 ± 116ab	98 ± 21abc	3.42 ± 0.6ab	0.0787 ± 0.0c
	T5	87 ± 33abc	18 ± 5.0bcd	86.98 ± 12ab	85 ± 13.7a	79 ± 29cde	4.76 ± 0.70a	0.0837 ± 0.0abc
	T6	78 ± 40abc	23 ± 13.2cd	80.45 ± 7.6ab	55 ± 7.2ab	62 ± 13cde	3.08 ± 0.4ab	0.0827 ± 0.0bc
	T7	79 ± 24abc	21 ± 3.06cd	85.14 ± 5.9ab	58 ± 1.1ab	70 ± 14cde	3.24 ± 0.0ab	0.0831 ± 0.0abc
	T8	65 ± 39bcd	19 ± 2.0cde	52.08 ± 58bc	72 ± 26abc	99 ± 46abc	4.03 ± 14ab	0.0848 ± 0.0ab
	T9	68 ± 46bcd	15 ± 2.0def	18.05 ± 65cd	84 ± 11.8a	64 ± 2.5cd	4.68 ± 0.6ab	0.0819 ± 0.0bc
	T10	54 ± 47bcd	12 ± 1.7def	84.95 ± 6.6ab	53 ± 14ab	99 ± 16abc	2.92 ± 0.7ab	0.0831 ± 0.0abc
	T11	74 ± 48abc	28 ± 7.02ab	10.48 ± 123d	52 ± 6.2ab	49 ± 21efg	2.89 ± 0.3ab	0.0846 ± 0.0ab
	T12	98 ± 28ab	31 ± 4.7ab	70.02 ± 9.1ab	37 ± 9.4e	98 ± 41abc	2.08 ± 0.5e	0.084 ± 0.0ab

a LFW = leaf fresh weight, LDW = leaf dry weight, LMC = leaf moisture content, LA = leaf area, LAR = leaf area ratio, LAI = leaf area index, and NAR = net assimilation rate.

b Values are the mean ± SD of plants from each treatment.

c The treatments exhibit dissimilar letters within rows that represent significance (*p* ≤ 0.05) level.

### 3.2 Determination of Physiological and Biochemical Attributes

**Figure 3:**
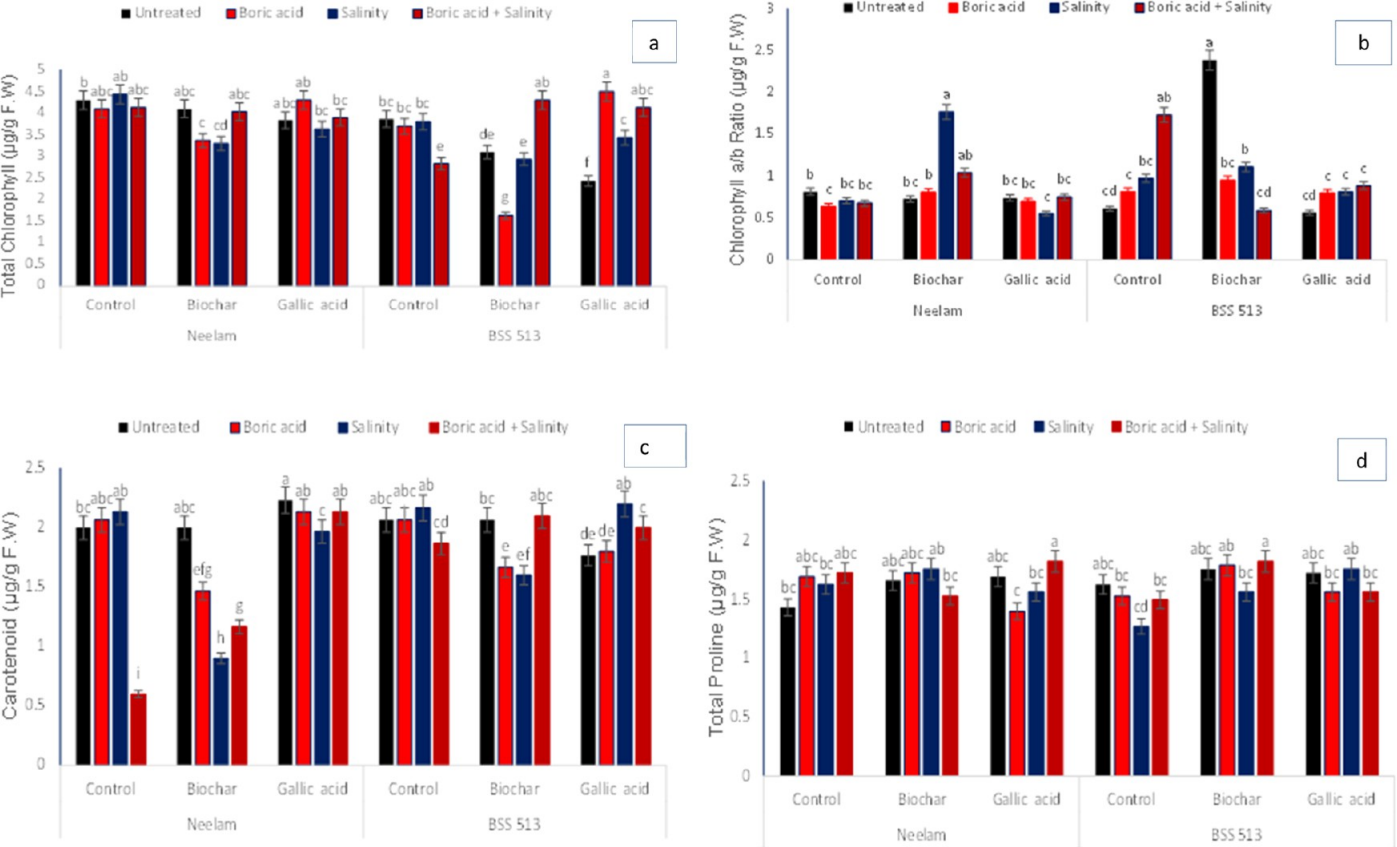
Effects of biochar (5 g kg^–1^) and gallic acid (2 mM) on the (a) total chlorophyll *a*, (b) chlorophyll *a*/*b* ratio, (c) carotenoid, and (d) total proline in foliar (mean ± standard) under induced salinily (120 mM NaCl) and boron (25 mg kg^–1^) stress. Vertical bars indicate for standard errors with least significance difference among mean values at *p* < 0.05.

The concentration of photosynthetic components, including the total chlorophyll content (TCC) and chlorophyll *a*/*b* ratio (CABR), revealed that salinity stress decreased TCC and CABR with biochar (T7) and GA (T11) treatment significantly (*p* ≤ 0.05) in Neelam, whereas in variety BSS 513, boron stress (T6) and combined salinity and boron stresses (T8) reduced TCC and CABR with biochar. A greater increase in TCC and CABR in Neelam was observed with gallic acid treatment under boron stress (T10) and biochar amendment to soil under salinity stress (T7). In addition, GA-treated seeds after exposure to boron stress (T10) and in biochar (T5)-amended soil exhibited enhanced TCC and CABR in BSS 513. ANOVA results revealed a positive interaction between GR, T × GR and G × T × GR at *p* < 0.05 (Figure 3 and Table 5).

Under salt and boron stress, the carotenoid content (CC) and TPC increased; the increase was greater with GA treatment and biochar application in both varieties. Thus, GA treatment (T9) and biochar-amended soil under applied salinity stress (T7) regulated CC and TPC in the variety Neelam. In contrast to variety BSS 513, GA treatment under salinity stress (T11) and biochar application under combined abiotic stress (T8) enhanced CC and TPC significantly. Likewise, a large reduction in CC under combined stress (T4) in both varieties and TPC was observed in boron stress (T10) in Neelam and salinity (T3) in the variety BSS 513. The interactions were significant (*p* < 0.05) between growth regulators, T × GR, and G × T × GR (Figure 3 and Table 5).

**Figure 4:**
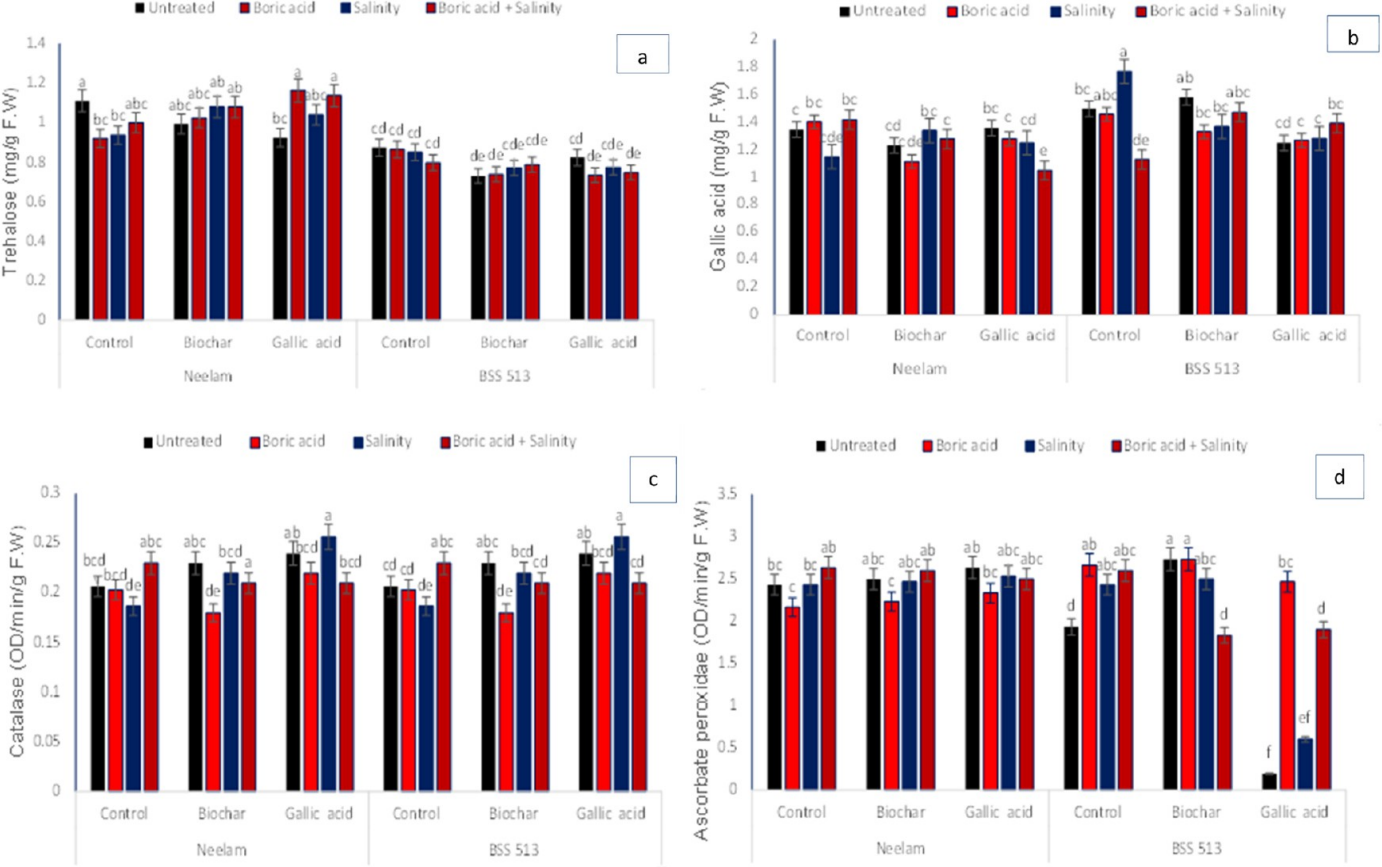
Effects of biochar (5 g kg^–1^) and gallic acid (2 mM) on (a) trehalose, (b) gallic acid, (c) catalase, and (d) ascorbate peroxidase in foliar (mean ± standard) under induced salinity (120 mM NaCl) and boron (25 mg kg^–1^) stress. Vertical bars indicate standard errors with least significance difference among mean values at *p* < 0.05.

The highest values of TS and GA of foliar material treated with gallic acid (T12) and without gallic acid (T4) under combined stress were observed in Neelam. In addition, GA non-stress-treated (T9) and combined abiotic stress-treated plants with gallic acid (T12) reduced TS and GA contents in the variety Neelam. However, an increase in the same parameters in the variety BSS 513 was observed with boron (T2) and salinity stress (T3), while a decrease in TS and GA was observed under biochar applied to soil (T5) and combined stress treatments (T4). The *F*-ratio revealed significant results in terms of interactions between genotype, growth regulators, G × GR, and G × T × GR (Figure 4 and Table 5).

To assess the effect of biochar and GA on resistance to boron and salinity stress, the activity of antioxidant enzymes in foliar plant materials was analyzed (Figure 4). GA pretreatment of seeds enhanced CAT in both varieties under salinity stress (T11), whereas low-level activity was observed under boron stress with biochar treatment (T6). In contrast, the APX content of Neelam increased with GA treatment (T9) and biochar amendment to soil under boron stress (T6) but decreased under boron stress (T2) in the Neelam variety and in plants treated with GA (T9) in BSS 513. ANOVA results revealed significant results for all interactions for APX (Table 5).

### 3.3 Determination of Stomata and Trichomes

**Figure 5:**
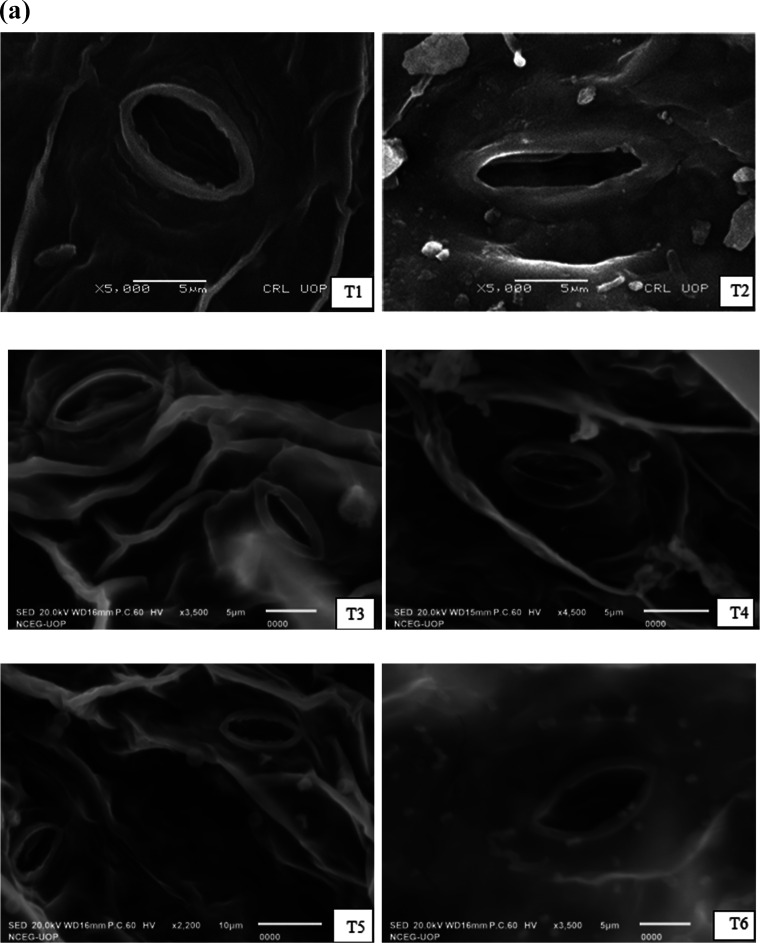

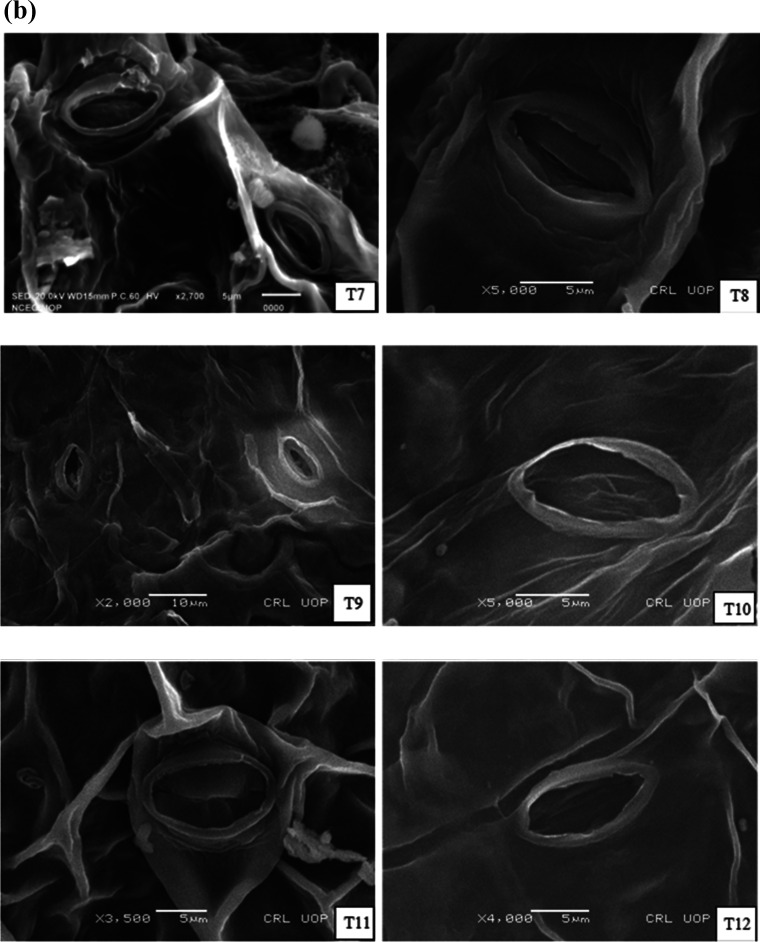

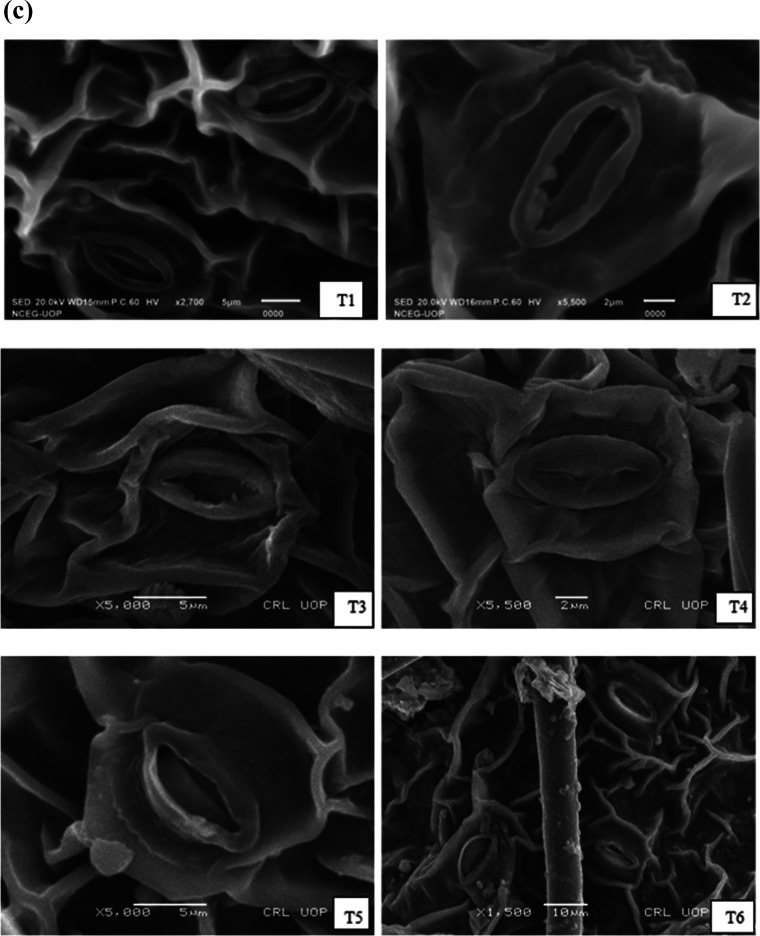

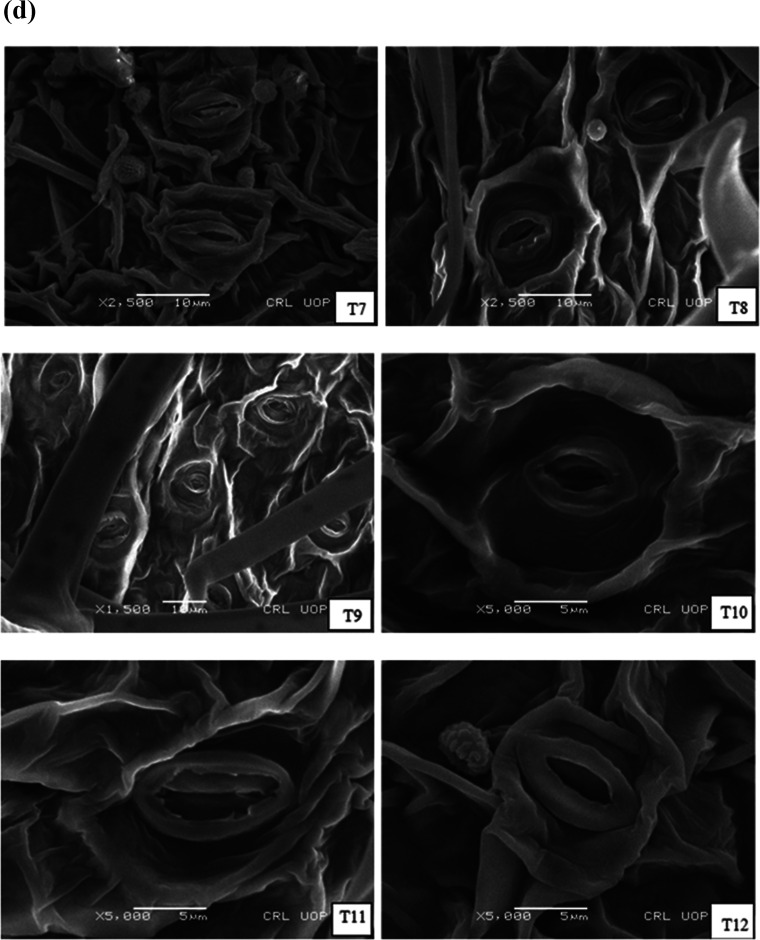
(a, b) SEM micrographs indicating changes in the leaf stomata size and physiology of the *S. melongena* L. variety Neelam under induced salinity (120 mM NaCl) and boron (25 mg kg^–1^) stresses. (c, d) SEM micrographs indicating changes in the leaf stomata size and physiology of the *S. melongena* L. variety BSS 513 under induced salinity (120 mM NaCl) and boron (25 mg kg^–1^) stresses.

Stomata modify their physiology to regulate water absorption by opening and closing along with the size, density, and turgidity of guard cells. The stomatal morphology and epidermal vigor of the susceptible variety Neelam was significantly (*p* = 0.05) affected by combined salinity and boron stress (Figure 5a,b). Sunken and partially and completely closed stomatal apertures with flaccid guard cells were observed under T8 and T12. The subsidiary cells appeared as clusters and somewhat dehydrated due to water insufficiency under osmotic stress. However, biochar and GA treatments significantly increased resistance to the toxic effect of individual abiotic stress and nonsignificantly under combined abiotic stresses. The stomata aperture and epidermal vigor of BSS 513 were also significantly affected by combined abiotic stresses under T4 and T8 (Figure 5c,d). Stomatal alterations in response to combined salinity and boron stress were observed as indicated by quenched and reduced stomatal aperture size and epidermal vigor. However, biochar and GA treatments significantly improved the physiology of stomata and subsidiary cells by opening stomatal apertures and increasing the stomata size, water potential of guard cells, and epidermal vigor, which increases resistance to abiotic stresses. SEM results indicate that biochar GA treatments improved the stomata size and physiology, guard cell turgidity, and epidermal vigor under individual and combined abiotic stresses.

**Figure 6:**
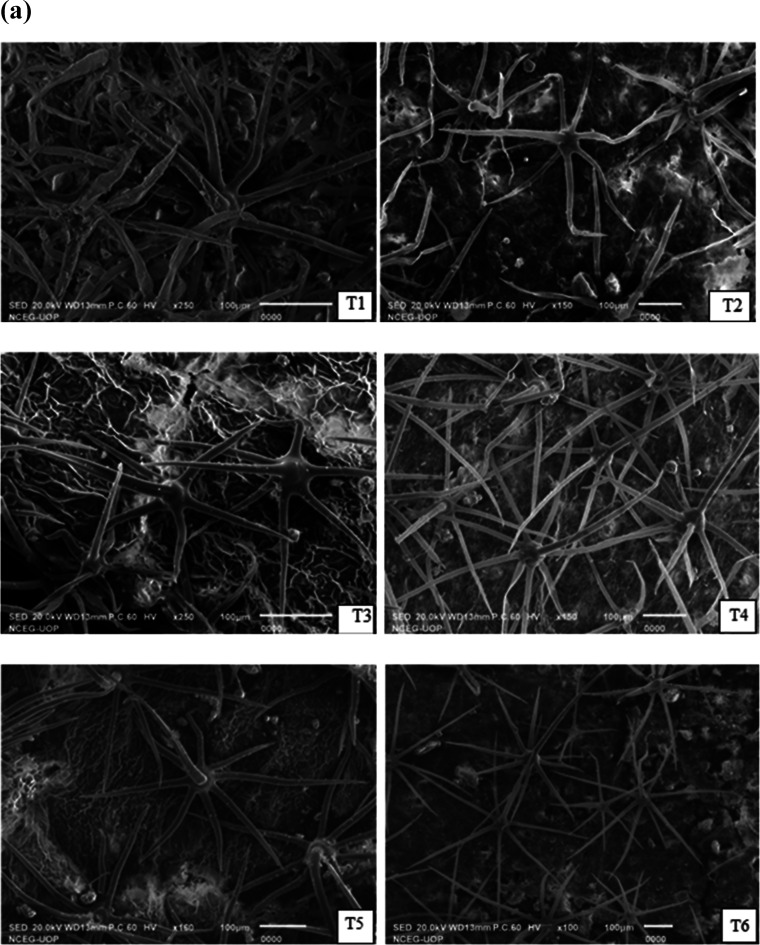

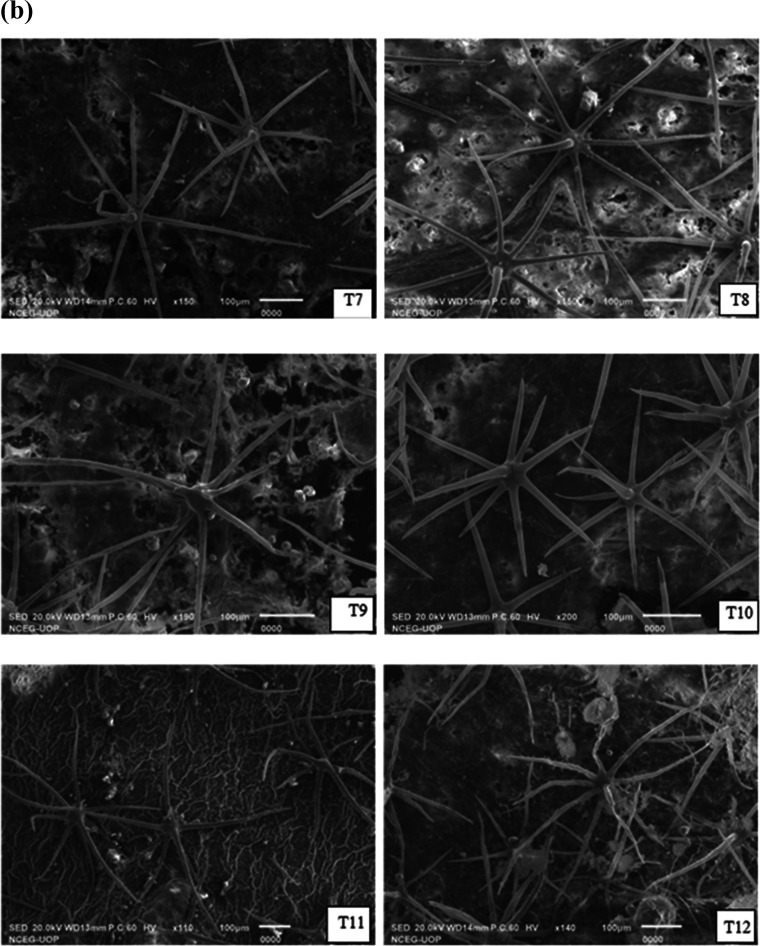

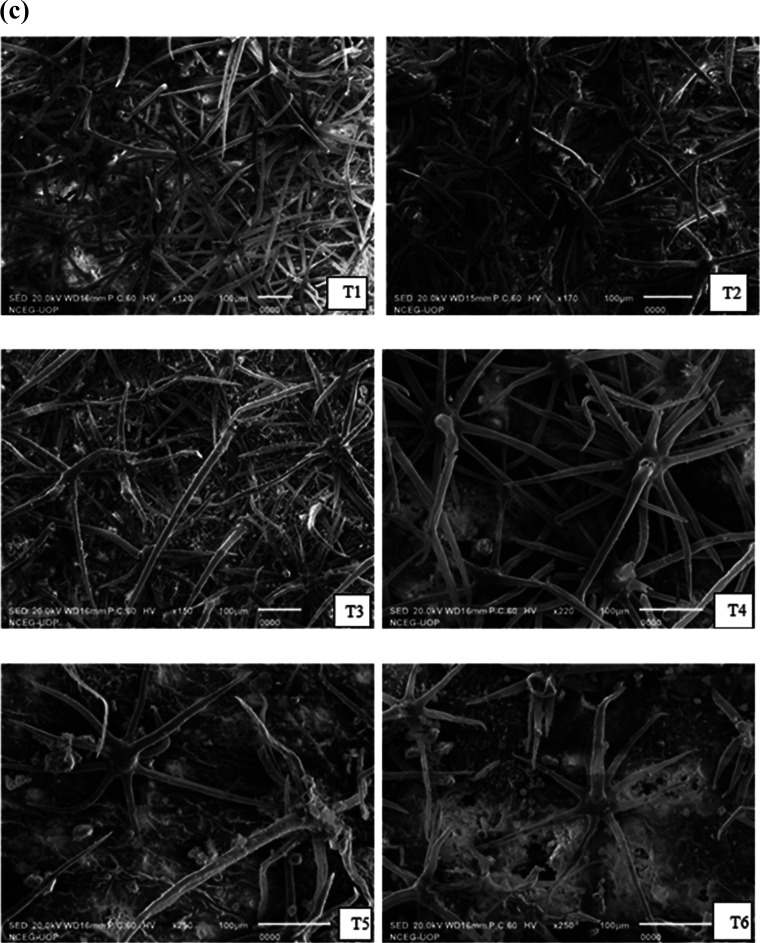

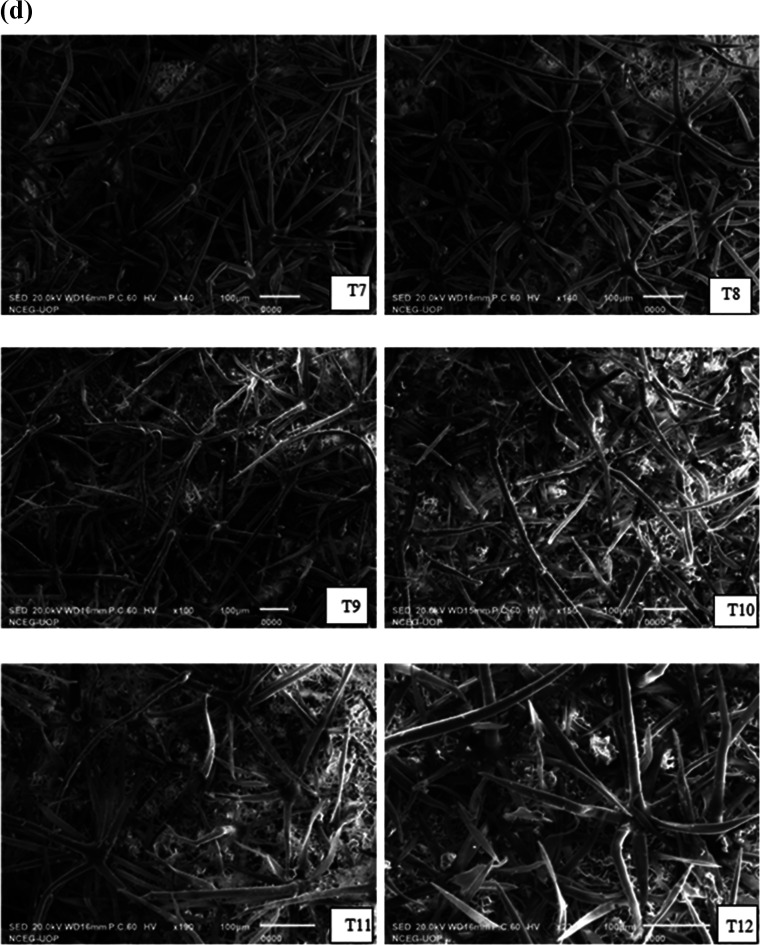
(a, b) SEM micrographs indicating changes in the trichome density of the *S. melongena* L. variety Neelam under induced salinity (120 mM NaCl) and boron (25 mg kg^–1^) stresses. (c, d) SEM micrographs indicating changes in the trichome density of the *S. melongena* L. variety BSS 513 through SEM under induced salinity (120 mM NaCl) and boron (25 mg kg^–1^) stresses.

SEM revealed nonglandular stellate-shaped trichomes with pointed ends with a size range from 270 to 3125 μm in length (Figure 6a,b and Table 3). Salinity stress decreased the average density of trichomes by 5.3 mm^2^. Treatment with GA significantly (*p* < 0.05) increased trichome density by 11 mm^2^ in Neelam under combined abiotic stress. Biochar alone (T5) reduced the trichome density by 5.0 mm^2^ in BSS 513, which was significantly improved by 23 mm^2^ with GA treatment when compared with the control (Figure 6c,d and Table 3). These results suggest that GA treatment under abiotic stress mediates plant molecular mechanisms that enhance the size and density of trichomes to conserve water and reduce osmotic stress inside the cell.

**Table 3:** Effect of Biochar (5 g kg^–1^) and Gallic Acid (2 mM) on Leaf Anatomical Characteristics of *S. melongena* L. under Induced Abiotic Stresses^a^^,^^b^^,^^c^

	SS (μm)		SI (1 mm)		UET (μm)		LET (μm)		CT (μm)	
treatment	Neelam	BSS 513	Neelam	BSS 513	Neelam	BSS 513	Neelam	BSS 513	Neelam	BSS 513
T1	17 589 ± 578bcd	475.0 ± 101.0b	21.10 ± 0.29a	25.4 ± 0.20bcd	64 ± 0.9abcd	21.0 ± 0.20g	57 ± 1.6bcde	14.0 ± 0.40g	4.80 ± 1.30f	5.20 ± 1.5c
T2	9989.0 ± 907cde	400.0 ± 76.00b	31.7 ± 0.96de	24.00 ± 0.50de	82.0 ± 3.2abc	32.0 ± 0.80d	93.0 ± 4.50b	26.0 ± 1.2de	5.6 ± 1.70ef	5.40 ± 1.5c
T3	1589.0 ± 16.00e	485.0 ± 174.0b	20.6 ± 0.410f	23.200 ± 0.67e	117.0 ± 2.4a	28.0 ± 0.80e	72.0 ± 3.6bc	30.0 ± 0.8cd	6.2 ± 1.90de	5.20 ± 1.5c
T4	299.00 ± 64.00e	334.0 ± 90.0b	18.0 ± 0.49b	19.80 ± 0.600f	69 ± 56abcd	08.0 ± 0.20i	65.0 ± 37bcd	12.00 ± 0.2g	10 ± 3.800a	6.1 ± 1.9bc
T5	442.0 ± 125.00e	741.0 ± 70.0b	28.9 ± 0.23de	35.400 ± 0.13a	21.0 ± 1.10d	23 ± 0.40fg	25.0 ± 3.2de	23 ± 2.50ef	8.3 ± 2.9bc	6.5 ± 1.4bc
T6	32 857 ± 1283ab	424.0 ± 72.0b	21.40 ± 1.2de	27.30 ± 0.560b	102 ± 9.006a	27 ± 0.20ef	153.0 ± 8.0a	33.0 ± 0.0bc	8.8 ± 3.20b	4.90 ± 1.3c
T7	3666.0 ± 623de	1460.0 ± 421b	22.10 ± 0.82b	26.30 ± 0.78bc	40 ± 8.10bcd	29 ± 0..8de	99.0 ± 7.70b	34.00 ± 2.0b	9.0 ± 3.00b	6.20 ± 2bc
T8	2833 ± 1312.0de	3836.0 ± 340b	28.80 ± 0.31c	25.40 ± 0.36cd	22.0 ± 1.80d	32 ± 0.20d	100 ± 8.10b	33.0 ± 0.3bc	11.0 ± 4.7a	5.10 ± 1.4c
T9	938.00 ± 242.00e	5146 ± 2483b	25.2 ± 0.60de	23.90 ± 0.58de	26.0 ± 2.0cd	15.0 ± 0.30h	31.0 ± 13cde	13.0 ± 0.20g	5.7 ± 1.8ef	7.5 ± 2.5ab
T10	39 246.0 ± 1425a	1050.0 ± 170b	20.90 ± 0.32e	19.40 ± 0.320f	96.0 ± 2.1ab	42.0 ± 0.8b	57 ± 1.6bcde	26.0 ± 0.8de	7.3 ± 2.5cd	6.6 ± 2.2bc
T11	13 381 ± 3478cde	796.00 ± 294b	20.20 ± 0.33d	18.6 ± 0.410fg	13.00 ± 0.2d	37.0 ± 1.2c	15.0 ± 0.40e	21.0 ± 1.60f	8.2 ± 2.8bc	5.40 ± 1.5c
T12	24 470 ± 6981abc	11346 ± 5044a	22.50 ± 0.36a	16.940 ± 0.69g	123.0 ± 0.8a	75.0 ± 2.8a	90.0 ± 0.80b	95.0 ± 0.80a	5.4 ± 1.0ef	9.0 ± 3.30a

a SS = stomata size (1 mm), SI = stomata index, UET = upper epidermis thickness (μm), LET = lower epidermis thickness (μm), and CT = cuticle thickness (μm).

b Values are the mean ± SD of plants from each treatment.

c The treatments exhibit dissimilar letters within rows that represent significance (*p* ≤ 0.05) level.

### 3.4 Determination of Leaf Anatomy

Leaf structure analysis (Table 3) of images from an optical microscope (DS-U3, Nikon, Japan) revealed that GA treatment (T10) significantly increased the stomata size by 13 381 μm under salinity stress in Neelam and by 11 346 μm under combined salt and boron stress (T12) in BSS 513. However, both stresses in combined form decreased the trait more negatively in treatment T4 by 299 μm. In contrast, the biochar treatment of soil significantly (*p* < 0.05) enhanced leaf SI in both varieties (range 28.9–35.4 mm^2^). Leaf SI decreased up to 18 mm^2^ with combined salt and boron stress (T4) without growth regulators in the Neelam variety and with growth regulators in BSS 513. Our results confirm the adverse effects of combined stress conditions on leaf photosynthetic aperture by decreasing the number of stomata per epidermal cell compared to gallic acid and biochar-treated plants.

Plants treated with GA under combined abiotic stress (T12) exhibited increased thickness of upper epidermis (UET) by 123 μm in Neelam and 75 μm in BSS 513. The thickness of lower epidermis (LET) was significantly improved by biochar treatment of soil by 153 μm in Neelam and 95 μm in BSS 513 for boron stress (T6) and combined abiotic stress (T12). However, salinity stress reduced UET and LET by 13 and 15 μm in the variety Neelam under T11, while in BSS 513, a decrease was observed by 0.8 and 12 μm in UET and LET under combined abiotic stress, respectively. Both biochar- and GA-treated plants exhibited markedly improved leaf morphology and resisted these abiotic stresses when compared with control (untreated) plants. Similarly, cuticle thickness (CT) was enhanced by 11.4 μm in Neelam and 9.0 μm in variety BSS 513 for T12 in both varieties after salinity and boron stress (T8) when compared with the control. However, boron stresses without and with biochar reduced cuticle thickness by a mean of 5.6–4.9 μm for T2 and T6 in the variety Neelam and BSS 513 (Table 4).

**Table 4:** Effect of Biochar (5 g kg^–1^) and Gallic Acid (2 mM) on Leaf Anatomical Characteristics of *S. melongena* L. under Induced Abiotic Stresses^a^^,^^b^^,^^c^

	TD (μm)		VIN (1 mm)		VTN (1 mm)		MT (μm)		LT (μm)	
treatments	Neelam	BSS 513	Neelam	BSS 513	Neelam	BSS 513	Neelam	BSS 513	Neelam	BSS 513
T1	5.6 ± 2.1de	9 ± 7.8ghi	13.0 ± 0.810a	10 ± 0.810bcd	17.00 ± 0.810a	14 ± 0.81bcd	3014 ± 107b	773 ± 33d	111 ± 13c	471 ± 1.60a
T2	7.0 ± 2bcd	12 ± 2.5ef	9.00 ± 0.810c	8 ± 0.810cdef	11.0 ± 0.81cde	10 ± 0.810ef	1399 ± 255d	471 ± 41fg	427 ± 16d	293.0 ± 79a
T3	6.6 ± 1.1cd	10.0 ± 2gh	8.3 ± 0.940cd	5.00 ± 0.810f	7.00 ± 2.100e	8.0 ± 0.810f	467.0 ± 10e	387 ± 20g	230 ± 53b	565.0 ± 14a
T4	10 ± 1.50ab	8.0 ± 3.6hi	7.3 ± 0.470cd	10 ± 0.81bcd	10.0 ± 1.60cde	17 ± 0.810ab	758.0 ± 92e	819 ± 1.6d	85.00 ± 3e	107.00 ± 2a
T5	6.6 ± 0.5cd	5.0 ± 1.7ij	10.0 ± 0.81bc	11.0 ± 0.81bc	13.6 ± 1.20abc	14 ± 0.81bcd	3286 ± 320b	482 ± 0.8f	62.00 ± 3e	67.0 ± 0.8a
T6	10 ± 1.1ab	9 ± 3.1ghi	6.0 ± 0.810d	5.00 ± 0.810f	7.00 ± 0.8100e	13 ± 0.81cde	3511 ± 193b	514.0 ± 2f	81.00 ± 6b	556 ± 4.30a
T7	4.00 ± 1.0e	16 ±± 1cd	12.0 ± 0.81ab	7.0 ± 0.81def	16.0 ± 0.810ab	11 ± 0.81def	2473 ± 157c	681 ± 15.0e	91.0 ± 16c	410 ± 4.40a
T8	5.3 ± 0.5de	17 ± 3.6bc	100 ± 0.810bc	6.0 ± 0.81ef	12 ± 0.810bcd	10.0 ± 0.81ef	1868 ± 10d	818 ± 0.8d	140 ± 24d	336 ± 5.00a
T9	6.3 ± 1.5cd	22 ± 2.1ab	13.0 ± 0.810a	14.00 ± 1.60a	10.3 ± 0.81cde	19.30 ± 1.60a	446.0 ± 5.3e	847 ± 1.6d	73.0 ± 2.0e	71.0 ± 1.20a
T10	9.6 ± 1.1bc	23 ± 7.50a	8.20 ± 0.81cd	12.0 ± 0.81ab	10.0 ± 0.81cde	16.0 ± 0.81bc	3218 ± 2.4b	1916 ± 54c	236.0 ± 2c	468 ± 1.20a
T11	9.6 ± 3.7bc	12 ± 2.50ef	9.00 ± 0.810c	11.0 ± 0.81bc	12.0 ± 1.60bcd	13. ± 0.81cde	5546 ± 7.9a	2399 ± 43b	153 ± 17a	736 ± 0.80a
T12	11.0 ± 6.1a	19 ± 1.1abc	8.0 ± 0.810cd	90 ± 0.81bcde	9.00 ± 0.810de	12.6 ± 0.47de	3322 ± 88b	2841 ± 0.4a	141 ± 1.2a	690 ± 2.40a

a TD = trichome density, VIN = vein islet number, VTN = vein termination number, MT = midrib thickness (μm), and LT = lamina thickness (μm).

b Values are the mean ± SD of plants from each treatment.

c The treatments exhibit dissimilar letters within rows that represent significance (*p* ≤ 0.05) level.

**Figure 7:**
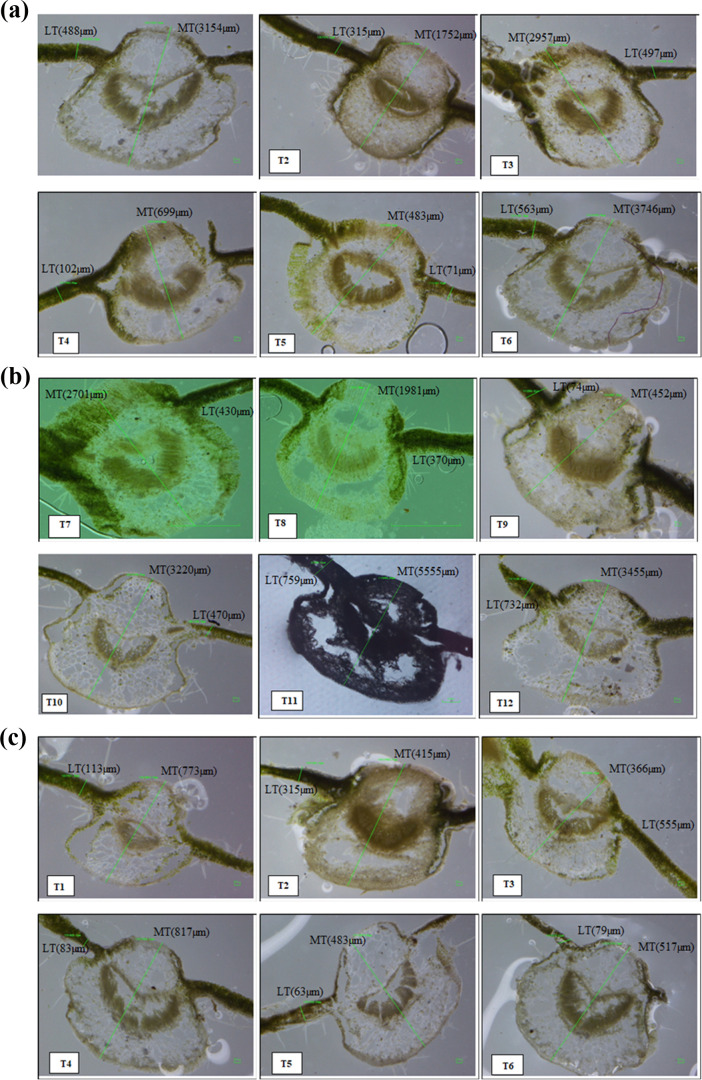

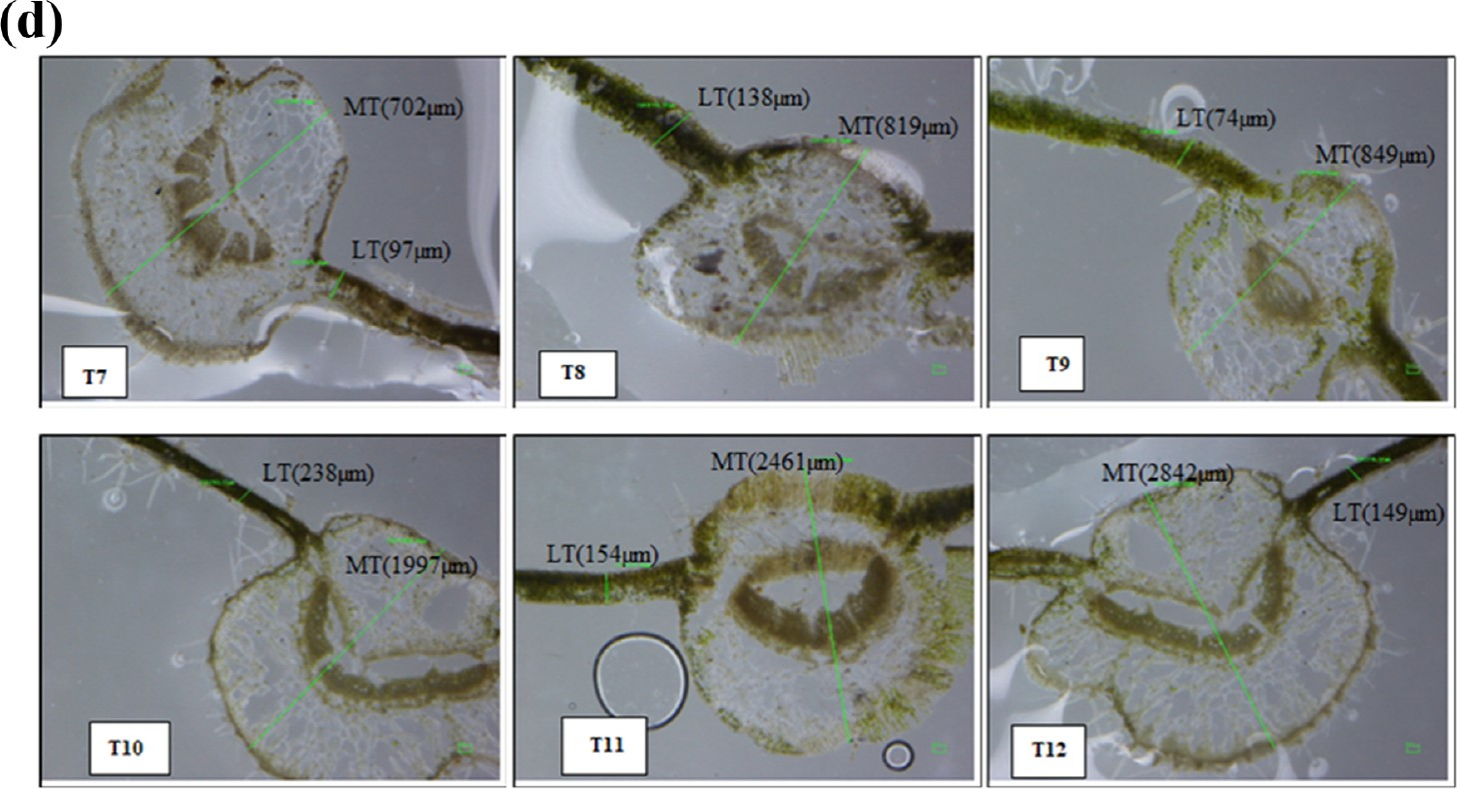
(a, b) Light micrographs indicate changes in the midrib thickness (MT) and lamina thickness (LT) of the *S. melongena* L. variety Neelam under induced salinity (120 mM NaCl) and boron (25 mg kg^–1^) stresses. (c, d) Light micrographs indicate changes in the midrib thickness (MT) and lamina thickness (LT) of the *S. melongena* L. variety BSS 513 under induced salinity (120 mM NaCl) and boron (25 mg kg^–1^) stresses.

The variation in the vein islet number (VIN) ranged from 13 per mm^2^ in Neelam to 14 per mm^2^ in BSS 513 with GA treatment (T9) and was reduced under salinity stress (Table 4). Our results indicate that the interactions of boron stress and biochar (T7) enhanced vein termination numbers (VTN) from 16 per mm^2^, which was the apparent minimum under salinity stress without applied growth regulators (T3), irrespective of the variety. In BSS 513, VTN was 19 per mm^2^ in GA-treated plants (T9). Moreover, GA treatment under salinity stress (T11) in Neelam and combined abiotic stress (T12) in BSS 513 significantly increased midrib thickness (MT) by 5546 and 2841 μm, respectively. Lamina thickness (LT) increased by 236 and 736 μm for boron stress (T10) and salinity stress (T11), respectively (Figure 7a–d and Table 5). Furthermore, LT decreased in both varieties with biochar amendment (T5) by 62–67 μm. Applied salinity stress lowered MT by 387–467 μm in both varieties. Micrographs revealed significant differences in leaf anatomical features between the two varieties.

**Table 5:** *F*-Ratio and Significant Level of Morpho-Anatomical and Physiological Attributes in *S. melongena* L.^a^

	source of variation						
trait	genotype (G)	treatment (T)	growth regulator (GR)	G × T	G × GR	T × GR	G × T × GR
SS	110***	25.3***	26.5***	33***	6***	12.9***	12***
SI	4***	193***	459***	60***	105***	111***	54***
UET	98***	21***	9***	6***	14***	18***	8***
LET	270***	53***	22***	18***	41***	28***	8***
CT	87***	13.9***	37***	13***	101***	16***	45***
LT	3.1	50***	27***	5.5***	27***	10***	8.5***
VIN	3***	50***	27***	5***	27***	10***	8***
VTN	40***	30***	3**	11***	26***	17***	18***
MT	1889***	354***	667***	258***	13***	266***	105***
TD	140***	47***	72***	44***	153***	69***	71***
LFW	22***	0.80	0.26	0.03	0.46	0.48	044
LDW	47***	6.1***	0.05	0.34	0.23	4.1***	5.1***
LMC	0.04	2.31	1.33	1.32	1.61	0.80	1.49
LA	33***	9.7***	2.2	4.9***	3.1*	2.3*	2.6*
LAR	5.1*	4.4***	2.4	4.1**	1.3	2.1	3.8***
LAI	63***	9.7***	2.2	4.9***	3.1*	2.3*	2.6*
NAR	0.4	0.6	1.3	0.9	1.6	1	1.1
RWC	15***	1	4**	0.6	8***	1.2	1
RSR	7.8***	8.3***	6.6***	8.6***	0.1	6.1***	3.1**
TCC	7.1**	0.9	3.5*	0.5	0.5	4.8***	2.1
CABR	1.6	0.4	3.6*	0.4	0.2	1.5	2.7*
CC	0.07	2.2	5.1***	3.1*	3.6*	3.3***	0.8
TPC	2.7	0.4	3.6***	1.2	1.9	1.2	2.1*
TS	92***	0.1	0.2	0.7	4.1*	1.3	1.7
GA	20***	1.9	6.6***	1.3	1.7	1.8	8.8***
PPO	9***	3.1**	0.1	1.2	11***	4.2***	3.4***
CAT	0.35	2.6	2.2	1.1	0.3	1.9	0.5
APOX	45***	7.6***	35***	21***	44***	9.5***	11***

a LFW = leaf fresh weight, LDW = leaf dry weight, LMC = leaf moisture content, LA = leaf area, LAR = leaf area ratio, LAI = leaf area index, NAR = net assimilation rate, SS = stomata size (1 mm), SI = stomata index, UET = upper epidermis thickness (μm), LET = lower epidermis thickness (μm), CT = cuticle thickness (μm), TD = trichome density, VIN = vein islet number, VTN = vein termination number, MT = midrib thickness (μm), LT = lamina thickness (μm), TCC = total chlorophyll content, CABR = chlorophyll *a*/*b* ratio, CC = carotenoid content, TPC = total proline content, TS = trehalose sugar, GA = gallic acid, CAT = catalase, APOX = ascorbate peroxidase. *, **, and *** significant at *p* = 0.05. *p* = 0.01, and *p* = 0.001 probability levels, respectively.

## 4 Discussion

Changes in climate conditions are the main source of abiotic stresses,^89,90^ which have adverse effects on agricultural lands.^91^ Such changes in climatic conditions are interlinked with each other in various aspects that negatively affect plant physiology.^92,93^ The studies presented here were focused on the mitigating effects of woody biochar of *V. nilotica* L. and GA on morpho-anatomical, physiological, and biochemical aspects of *S. melongena* L. under induced salinity and boron stress. Biochar is generally a carbon-rich compound that resists decomposition when applied to soil and increases EC, pH, and crop yield. Biochar also reduces nutrient leaching from soil.^94^ However, Biochar–Na action mechanisms extend from basically no explanation to broad advantageous action or exchange Na^+^ ions with biochar-borne Mg^++^ and Ca^++^ ions. This electronegativity effect sorbs base cations such as Na^+^ to a higher level than other base cations (Ca and Mg) because of large radii and weak hydration soft base cations (Na); other base cations which are hard (Ca and Mg) may generate weak cation−π electron action due to their greater hydrated energies and thus have less efficaciously sorbed via biochar-delocalized π electrons.^94^ SEM and EDX analysis in the present study revealed more cracks and the porous nature of biochar with high EC and pH, improved cation exchange capacity, and high carbon/oxygen ratio. Hao et al.^95^ and Méndez et al.^96^ analyzed large pore size with cracks generated due to high-temperature pyrolysis that enhanced carbon stability in soil. Ahmad et al.^97^ and Kim et al.^98^ observed high bulk density and cation exchange capacity with an increased carbon/oxygen ratio. These studies are also in agreement with the findings of Lalay et al.,^74^ as the same keV of elemental peaks exposed the nutritional availability of various elements to crops that may be a possible approach to improve growth and productivity.

Disruption in plant growth and physiological activities of are primarily due to abiotic stress.^99,100^ These disturbances may occur when the plant is exposed to physical dehydration and the subsequent osmotic effects that block the passage of water in xylem vessels via ion toxicity.^101^ We observed a significant reduction in LDW, LAI, and LAR and a nonsignificant decrease in LFW and LMC under boron and salinity stresses. Growth attributes were significantly improved with biochar and GA treatments. Ibrahim et al.^102^ and Kanwal et al.^103^ revealed that salinity stress (150 mM) with 2 and 5% biochar application improved the leaf moisture content, leaf area, leaf fresh mass, and dry mass by 16, 16.5, 26.2, and 27.4%, respectively. In addition, salinity and salinity combined with boron stress led to a significant increase in the root–shoot ratio, which was then reduced by biochar amendment to soil. These results are consistent with the findings of Khan et al.^104^ and Shi et al.^105^ Chlorophyll is the main photosynthetic component of plants and is affected by ROS, including H_2_O_2_ and O_2_^–^, which cause lipid peroxidation and thus degeneration of chlorophyll.^106^ We observed a decline in chlorophyll content under stress regimes, which was improved by GA treatment. Taffouo et al.^107^ and Manuchehri and Salehi^108^ observed greater osmotic potential in stressed plants due to the elevated salt content, which significantly degraded and inhibited photosynthetic pigments. Parallel to our results, García-Sánchez et al.^109^ observed a pronounced reduction in total chlorophyll content due to salinity stress, which was ameliorated by GA and β-carotene application. The total content of chlorophyll in leaves was increased by 10% biochar, and the lowest was recorded in 12 dS m^–1^ salinity without biochar.^110^ Similar findings were described in seeds of rice primed with GA and rutin^66^ and through biochar in *Syngonium podophyllum*.^111^

Osmoprotectants, such as carotenoids, proline, and trehalose, are synthesized and accumulated in plant cells as a defense against hyperosmotic stress.^112^ As carotenoids, proline and trehalose play a defensive role against abiotic stresses in plants. Bhamburdekar and Chavan^113^ observed a reduction in carotenoid, proline, and trehalose contents under salinity and combined abiotic stresses. According to their findings, carotenoid and proline contents decreased under salinity and boron stresses in chickpea and wheat leaves. We observed that biochar and GA treatment enhanced the carotenoid and proline contents in *S. melongena* L. under abiotic stresses. Akladious and Mohamed^114^ and Linić et al.^115^ reported the highest level of carotenoids and proline in *Capsicum annuum* and Chinese cabbage with phenolic acid application under salinity stress. As an osmoprotectant, trehalose plays an important physiological role in maintaining the cellular osmotic balance and protects biological molecules under abiotic stress conditions.^116^ On exposure to salinity stress, we observed a reduction in trehalose content, which was improved by biochar and GA treatment under combined salinity and boron stresses. Our results are consistent with the previous work of Mehdizadeh et al.,^117^ who revealed that use of biochar enhances sugar levels by increasing NaCl. Similar results were also reported by Khodary,^118^ who suggested that the use of phenolic acid may trigger sugar metabolism by disrupting the enzymatic system for hydrolysis of polysaccharides under high osmotic pressure.

In natural environments, phenolic compounds are associated with scavenging systems for quenching ROS and therefore protect plants against tissue oxidation from free radicals.^83,111^ In the present study, the lowest GA levels were observed under saline conditions without growth regulators. Nevertheless, the opposite trend was observed under high boron concentration and combined abiotic stress through GA seed priming. The results are consistent with those of Tavallali et al.^119^ Lim et al.^120^ observed that accumulation of phenolic compounds was primarily due to rutin, isoorientin, and orientin. Babaei et al.^121^ observed that β-carotene and GA treatment of seeds enhanced levels of phenolic compounds upon exposure to salinity stress, consistent with our results. Consequently, higher antioxidant enzymatic activities likely provide greater resistance under abiotic stress conditions.^122^ The current study revealed that catalase and ascorbate activities increased after GA treatment under salt stress and decreased under boron stress. These outcomes are consistent with Babaei et al.,^121^ who observed that GA can participate in H_2_O_2_ detoxification by enhancing the activity of various antioxidant enzymes, including CAT, APX, SOD, and GPX in *Lepidium sativum* L. Similarly, Singh et al.^66^ also observed low H_2_O_2_ levels in plants treated with GA when compared with the control. Similar studies on the beneficial role of GA in improving antioxidant mechanisms in plants under salinity and boron stress have been described by Gunes et al.^57^ in *Vitis vinifera* and Ferro et al.^123^ in *Solanum esculentum*.

Leaf anatomical characteristics and internal leaf elemental compartmentalization were examined to elucidate relative salinity tolerance mechanisms in the varieties Neelam and BSS 513, which have superior salinity tolerance relative to other genotypes of the same species in prior screenings. SEM investigations of stomatal physiology revealed that leaves of *S. melongena* L. were sensitive to induced salt stress compared with boron stress and led to a considerable reduction in leaf size and density. Abnormal subsidiary cells may be due to osmotic stress, as salinity stress led to water shortage in these cells. Investigations with SEM by Torabi et al.^124^ and Orsini et al.^125^ revealed lower stomatal density, transpiration rate, and conductance as a means to resist salt stress in strawberry and borage. Our results indicate that modification in stomatal status had a significant effect on its movement and density and particularly turgidity of guard cells in plants after GA treatment. Consistent with this study, Ma et al.^126^ observed that treatment with phenolic acid reduced the negative impact of salinity stress under 0.3 and 0.6% NaCl treatment on stomatal and nonstomatal factors by enhancing stomatal density, thus improving photosynthetic ability in *Dianthus superbus* L.

Trichomes are epidermal cell developments in aerial parts of plants that facilitate responses to abiotic stress.^127^ SEM analysis on the density of covering trichomes on the abaxial epidermis in plants after GA treatment revealed an increased level of trichome density in both varieties but were decreased under abiotic stresses. Similar findings were obtained by Torabi et al.,^124^ where SEM micrographs revealed a reduced density of covering trichomes on the abaxial epidermis in plants exposed to salinity stress when compared with control plants. These outcomes were further corroborated by the results of Passinho-Soares et al.,^128^ who reported that growth regulators influenced quantitative and qualitative profiles of trichome distribution on the leaf surface. Bose et al.^129^ observed that growth regulators have a major role in trichome development. Likewise, Chakraborty^130^ observed that priming of tomato seeds with phenolic acid led to modified physiological attributes, including the seed vigor index, trichome density, and lengths of the shoot and root.

Leaf plasticity and changes in the morphological structure modulate both physiological and biochemical attributes in plants.^87^ In the present study, optical microscopy revealed an increase in SS and SI after biochar application in soil; a pronounced decrease was observed under salinity and boron stresses. Our results are consistent with previous work on tomato plants,^131^ which revealed that stomatal aperture and density were reduced under salinity stress. Similar findings were obtained by Akhtar et al.,^132^ who observed that biochar application improved both the stomatal aperture and density. A significant reduction in UET and LET were observed in stressed plants; this reduction was not observed with biochar application and GA treatment. These results are consistent with the observations of Hafez et al.^133^ in barley under stress and Leite et al.^134^ in *Astronium fraxinifolium*. Cutin is a polyester formed by hydroxy and hydroxy–epoxy (C16 and C18) fatty acid monomers^135^ that play a key role in reinforcing cuticle layers in leaves and roots to prevent water loss and thus maintain cellular turgidity. We observed improved cuticle thickness under combined stresses with applied biochar and GA compared with control groups. These results are consistent with those of Ndiate et al.,^136^ who observed formation of fatty acids in cuticle production in plants under stress conditions after biochar application. Moreover, the greater abundance of functional enzymes associated with production of medium chain fatty acids confirmed that biochar enhanced production for biosynthesis of the β-oxidation pathway and the fatty acid pathway for cutin production.^137^

Vein islet is a small photosynthetic tissue encircled by an area of conducting channel where the end terminal of the vein is veinlet termination points per millimeter on the surface of the leaf. Light microscopy revealed decreased VIN, VTN, MT, and LT under abiotic stresses, which were improved after biochar application or GA treatment in both varieties. Tak and Kakde^138^ observed a significant decrease in vein islet and termination number when tree foliage was exposed to pollutant stress. Our results confirmed the findings of Darwish et al.,^139^ who observed that riboflavin minimized the toxic effects of salt stress in tecoma plants, which reduced midrib and lamina thickness.

## 5 Conclusions

Biochar treatment of soil enhanced soil fertility and pH and maintained the moisture content. In addition, biochar application and GA treatment improved leaf parameters, chlorophyll content, osmoprotectants, phenolic compounds, and concentrations of antioxidant enzymes in *S. melongena* L. under salinity and boron stresses either individually or combined. SEM and light microscopy indicated that both biochar application and GA treatment alleviated the adverse effects of NaCl and boron toxicity by improving leaf morphology, epidermal vigor, and stomatal regulation. Moreover, a thick epicuticular layer and trichome density facilitated resistance to these stresses. The present study also revealed the susceptible nature of Neelam and the greater resistance of BSS 513 to these stresses. Given the conditions created by climate change, fulfilling the food demands of the rapidly increasing global population is a challenge for the scientific community. The results of this study suggest that application of growth regulators and employing priming techniques may open a new avenue to a sustainable agriculture by enhancing crop production and improving their abiotic stress resistance.

## Data Availability

All data generated or analyzed during this study are included in this published article.

## Notes

The seeds of two varieties (Neelam & BSS 513) of *S. melongena* L. were collected from the National Institute of Food and Agriculture (NIFA), Pakistan. All of the experiments were performed in accordance with relevant guidelines and regulations.

## Abbreviations

LDW: leaf dry weight
LFW: leaf fresh weight
LA: leaf area
LAI: leaf area index
LMC: leaf moisture content
RWC: relative water content
SEM: scanning electron microscopy
SS: stomata size
SI: stomata index
UET: upper epidermis
LET: lower epidermis
CT: cuticle thickness
TL: trichome length
VIN: vein islet number
VTN: vein termination number
CAT: catalase
APX: ascorbate peroxidase
